# E-learning in medical education: a perspective of pre-clinical medical students from a lower-middle income country

**DOI:** 10.1186/s12909-024-05158-y

**Published:** 2024-02-20

**Authors:** Uzair Abbas, Memoona Parveen, Falak Sehar Sahito, Niaz Hussain, Sundas Munir

**Affiliations:** 1 Dow University of Health Sciences, Karachi, Pakistan; 2 Liaquat University of Medical and Health Sciences, Jamshoro, Pakistan

**Keywords:** Medical education, Student’s perception, Online learning

## Abstract

**Background:**

Many of the educational institutions in developed countries have shifted to online learning. While transition from traditional to electronic learning (e-learning) has remained a great challenge in low-middle income countries, where limited resources for teaching and learning are important factors. Medical education involves not only lecturing but also deep understanding through laboratories and patient exposure. The debate about the effectiveness of e-learning in medical education is still in contradiction due to its limitations. This cross-sectional survey was conducted to assess pre-clinical undergraduate medical students’ perception of their first online learning in a lower-middle income country.

**Methodology:**

The survey was conducted among the students who had participated in online learning during COVID-19 for at least a year. A total of 824 preclinical medical students who completed the survey from public and private medical universities in Sindh, Pakistan were included in the study. We used a validated online-based questionnaire, distributed through E-mail and social media platforms to assess the perception of students regarding their first online learning experience.

**Results:**

The response rate of the survey was 87.9%. The mean age of students was 20.7 ± 3.8 years. 392/824 (47%) were males and 57% were females. Our study indicated that 613/824 (75%) of students were experiencing online learning for the very first time while 631/824 (77%) were facing technical issues like internet accessibility and lack of IT-related skills. 381/824 (46%) were not satisfied with the institute’s readiness for online teaching. However, 79% (654/824) of participants were of the idea that traditional learning is more effective in developing their practical skills as compared to e-learning. Of note, 668/824 (81%) showed overall dissatisfaction with e-learning.

**Conclusion:**

Based on our study findings, we concluded that most students have a negative perception of e-learning. Difficulty in connectivity, electricity issues, less interaction with colleagues and teachers, and issues with the structure of online courses were the most frequently reported problems by the students.

## Background

The current medical education curriculum emphasizes the development of a diverse set of professional skills that include a robust theoretical foundation, proficient clinical competencies, and effective interpersonal aptitudes, all of which are predominantly imparted through conventional teaching methodologies [1, 2]. Before COVID-19 pandemic, the method of learning used in various medical schools in Low-Middle Income Countries (LMICs) was mostly traditional in which face-to-face lectures were given in a classroom [3]. The COVID-19 pandemic significantly disrupted teaching in a variety of institutions. In many countries, including Pakistan, typical face-to-face classes had to be suspended to ensure the safety of students and teachers. To minimize the impact, medical schools had to find another approach to teach and, technology-based e-learning was the only option [4].

E-learning or online learning is a method of acquiring knowledge by using information technology [5]. The success of e-learning depends on many factors, including accessibility, usage of appropriate method**s**, course content, and assessment criteria [6]. E-learning, like any method of teaching, has its advantages and disadvantages for both student**s** and teachers. The benefits of e-learning that are worth mentioning include increased convenience and access to resources regardless of location and time [7]. Online classes also have limitations, including problems with internet access, poor internet connection quality, and insufficient digital skills of the participants including students as well as teachers [8]. Furthermore, the effectiveness of such an educational system is questionable, especially in the field of medicine where group discussions and peer interactions are necessary for knowledge and skill development [9, 10]. However, concluding the effectiveness of online and offline education is much more difficult and has failed to conclude [11]. In high-income countries, many academic institutions are using e-learning but in limited-resourced countries, adapting e-learning requires many adjustments to be made to make sure the e-learning is as effective as possible [12].

The global educational landscape has witnessed a profound transformation due to the COVID-19 pandemic. Many academic institutions that had previously been hesitant to advance their traditional pedagogical approach, were left with no choice but to completely switch to online teaching and learning [13]. Many institutes encountered various obstacles and hurdles in implementing e-learning in their institutes. The main difficulties encountered during online learning in LMICs were lack of prior experience, fewer IT resources, insufficient infrastructure, availability of the internet, and a lack of computer availability to all teachers and students [14, 15]. Medical institutes across Pakistan also confronted multiple challenges while adopting e-learning [16, 17].

Moreover, it is important to consider the student’s perception on a larger scale, regarding their e-learning experience to help finding the gaps in implementing online learning in the future in countries with low or limited resources. This study was conducted to evaluate the pre-clinical medical students’ perception regarding their online learning experience across Karachi Pakistan. This study also highlights the challenges faced by students during their first experience of online learning.

## Methodology

### Study design and setting

This cross-sectional study was conducted at Dow University of Health Sciences (DUHS) Karachi Pakistan between the period of July 2022 to July 2023 following ethical approval from the Institutional Review Board (IRB) of the university.

### Study participants

The target participants consisted of pre-clinical students of medical sciences (MBBS), Dental sciences (BDS), and allied health sciences (including bachelor programs in nursing, public health, and medical technology), from public and private medical universities across Karachi Pakistan.

### Inclusion and exclusion criteria

We included the students in the first and second year of above mentioned undergraduate degree programs who experienced online learning during 2021 and 2022 for at least a year during their academic sessions. The study excluded students who had progressed to their clinical rotation, and individuals who had never participated in online classes.

### Exposure to online learning

The students were taught online through a virtual learning environment (VLE) and Zoom application. The subjects taught during their initial year were Anatomy, Physiology, Biochemistry, and Pathology with additional subjects related to their degree programs.

### Sample size and data collection

**Fig. 1:**
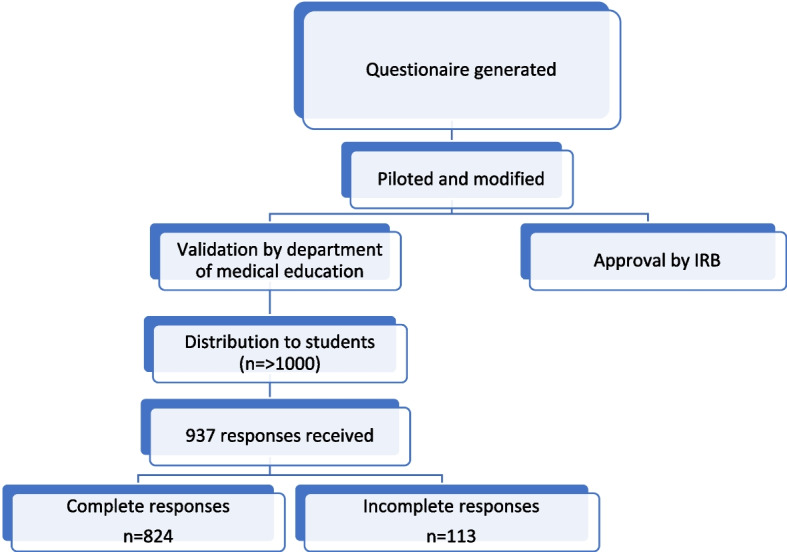
Steps of data collection. Figure shows the steps of generation of data collection tool, validation, and process of data collection

A non-probability purposive sampling technique was employed to select participants [18]. The sample size of the study was estimated from a reference study [19]. The questionnaire in Google form format was forwarded to the department of medical education of various universities and the responses were collected. We received 937 responses from the students however only 100% complete responses to all items of the data collection tool were included in the final analysis which were 824 to avoid any potential confounders and bias (Fig. 1).

### Data collection tool

A pretested, self-constructed questionnaire was developed using Google Forms. To safeguard anonymity, the identities of the participants were held in strict confidence. The questionnaire was piloted on 12 participants out of the study participants and modifications were made after suggestions. The final questionnaire was validated by the Department of Medical Education of our university and approved by the Institutional Review Board.

The data collection tool was comprised of a total of 31 items divided into 5 sections including demographics, and perception of experience of online learning, perception of participation in class activity during online sessions. The questionnaire also included perceptions regarding the institute’s preparedness and experience with online exams.

### Data analysis

The data obtained from the respondents was analysed using Microsoft Excel. Frequencies and percentages were presented in tabulated form. The comparison of perception between public and private medical institutes was performed on SPSS version 22.0 by applying Pearson’s Chi-square test.

## Results

### Demographic characteristics

We included 824 participants in our study. The mean age of students was 20.7 ± 3.17 years. 392/824 (47%) were males. There were 622 (75%) of students from public sector institutes while 202 were from private medical colleges. A total of 474/824 (57.5%) students were from MBBS, 212 (26%) from BDS, and 318 (37.5%) from allied health science programs (Table 1).

**Table 1:** Demographic characteristics of study participants *N* = 824

Variables		no.(%)
Age (years)	Mean	20.7 ± 3.17
Gender	Male	392 (47%)
	Female	432 (53%)
Institution type	Public	622 (75%)
	Private	202 (25%)
Field of education	Medical sciences	474 (57.5%)
	Dental sciences	212 (26%)
	Allied health sciences	318 (37.5%)

The table shows the mean age and gender of study participants, type of institute and their field of study.

### Perception of experience with online learning and participation in class activities

We asked about experiences and problems related to online learning. 613/824 (75%) students were experiencing online learning for the first time. 674 (82%) were facing technical problems during online classes like internet connectivity and electricity supply and only 460 (55.5%) were able to attend classes regularly.

A larger number of students 512 (62.5%) were unable to actively participate during online learning. However, 50.5% experienced proper engagement during classes while 721(87%) were of the idea that they get easily distracted during an online class. Among 824, 680/824 (82%) agreed that having a teacher in the classroom is necessary for their learning. In our survey, we found 536/824 (65.5) of participants were not able to interact with their colleagues and teachers during the online sessions (Table 2).

**Table 2:** Perception of students on experience with online learning and participation in class activities

Sections and questions	Response in YES n (%)	Response in NO n (%)
**Section I: Perception of Experience with Online Learning**		
** I have experience of attending online courses in the past**	211 (25%)	613 (75%)
** I have the proper equipment for attending online classes**	689 (84%)	135 (16%)
** I have a good command of computer skills**	511 (62%)	313 (38%)
** I face technical problems during online classes like internet connectivity and electricity issues.**	674 (82%)	150 (18%)
** I can take online classes regularly**	418 (50.5%)	406 (49.5%)
**Section II: Perception of Participation in Class Activities:**		
** I can fully participate in online classes actively**	312 (37.5%)	512 (62.5%)
** I feel engaged during online class**	418 (50.5%)	406 (49.5%)
** I get distracted during online class**	721 (87%)	103 (13%)
** There is proper student activity during online class**	692 (83%)	132 (17%)
** For me, home is a better place to learn than to classroom**	221 (26%)	603 (61%)
** For me, having a teacher in the classroom is necessary while learning**	680 (82%)	144 (18%)
** I was able to interact with my colleagues and teachers during online class**	288 (34.5%)	536 (65.5%)
** I have proper access to online learning material**	456 (55%)	368 (45%)

The students were asked about their perceptions regarding their experience with online learning and participation in class activities. The response was collected as YES or NO.

### Perception of the Institute’s preparedness and online exams

We then asked about the satisfaction of students regarding their institutions’ preparedness for the sudden shift to online classes. For access to online learning material, 456 (55%) had proper access to that while 512 (64.5%) were not satisfied with the structure of online courses as compared to traditional learning methods. More than 70% of students complained about unresolved queries regarding topics and inability to develop clinical and laboratory skills in online classes as compared to traditional face-to-face learning. Overall, 502 (61%) of students were not satisfied with the institute’s preparedness regarding online teaching (Table 3).

**Table 3:** Perception of students towards institute’s preparedness and online exams. (*n* = 824)

Sections and questions	Response in YES n (%)	Response in NO n (%)
Section III: Perception of Institute’s Preparedness:		
Course objectives and curriculum were well explained to me	444 (53.5%)	360 (46.5%)
Learning material was provided properly	502 (60%)	322 (40%)
My online course was highly structured as the traditional course used to be	294 (35.5%)	512 (64.5%)
All my queries regarding the subject were resolved during online classes	236 (28.5%)	588 (71.5%)
Online learning has helped me to develop clinical and lab skills more than traditional learning.	170 (20.5%)	654 (79.5%)
I was able to complete my tasks/ assignments on time	510 (61%)	314 (39%)
Overall, I am satisfied with the institution’s readiness for online teaching	322 (39%)	502 (61%)
Section IV: Perception of online exam:		
I appeared in an online exam/ class test (both theory and viva)	650 (79%)	174 (21%)
I faced technical problems while attempting an online exam	514 (63%)	310 (37%)
The online exam was well-structured	616 (74.5%)	208 (25.5%)
I was able to complete my exam in the given time	627 (76%)	197 (24%)
I was able to interact with the examiner during the online viva exam	448 (54%)	376 (46%)
Overall, online learning is more effective than traditional learning.	156 (18%)	668 (81%)

About 79% of participants were able to appear in the online exam while 514 (63%) faced connectivity issues while attempting online exams. Moreover, 448/824 (54%) were unable to properly communicate with the examiner during the online viva exam (Table 3).

The students were asked about their perceptions regarding the institution’s preparedness and online exam experience. The response was collected as YES or NO.

### Important factors highlighted from students’ perception of e-learning

**Fig. 2:**
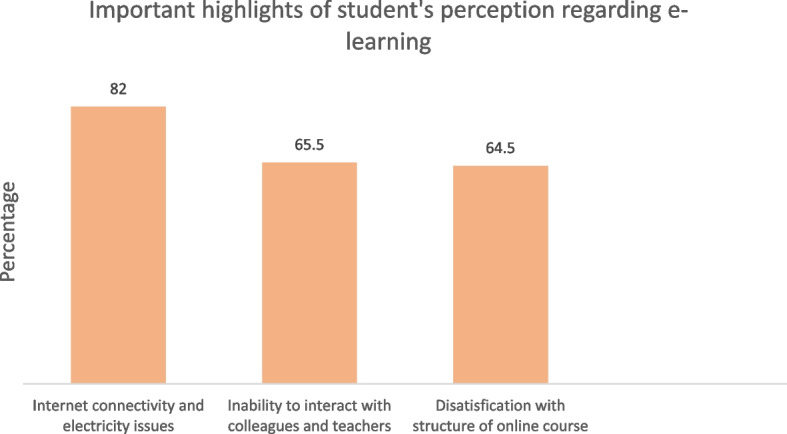
Important highlights of student’s perception regarding e-learning. *N* = 824. The figure shows the percentage of students’ responses

We observed that 674 (82%) of students were facing internet connectivity and electricity issues to connect for online learning while 536 (65.5%) were unable to interact with colleagues and teachers during online learning. Moreover, 512 (64.5%) were not satisfied with the structure of online learning (Fig. 2).

### The overall perception of online learning

**Fig. 3:**
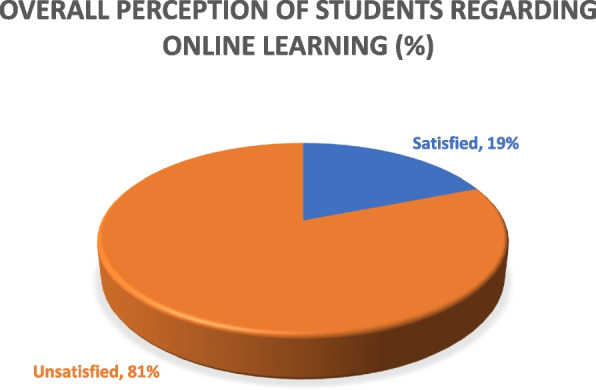
Overall perception of medical students regarding online learning experience *n* = 824

Of note, only 156 (19%) of students were satisfied with the online learning method however 668 (81%) of the students were not satisfied with the online learning method in our study (Fig. 3).

### Comparison of perception of students regarding e-learning from public and private medical institutes

We further evaluated the difference in perception of medical students from public and private sector medical institutes. We had 202 (25%) and 622 (75%) participants from private and public sector medical institutes respectively. Pearson’s Chi-square was applied to observe any difference in perception based on private or public sector medical students. Of all 30 items of our data collection tool including age, gender, and other parameters, we did not find any significant difference in the perception of students regarding e-learning based on their type of institutes (data not shown; p= > 0.05; CI = 95%).

## Discussion

We found that 81% of participants were not satisfied with the e-learning experience, which is the highest reported number to date. The most frequently reported issues were internet connectivity and electricity problems to connect for online learning (82%), no proper interaction with colleagues and teachers during online learning (65.5%), and problems with the structure of online courses (64.5%). Technical issues in connectivity for online learning (Internet inaccessibility and unavailability of electricity) remained a critical concern with a significant portion of the students in our study. As reported by Aristovnik et al., the primary issue in online learning in LMIC is poor internet connectivity and electricity supply issues which are concordant with our study [20]. We found, 50% of students were not able to take online classes regularly. Bediang et al. reported the same as a major barrier to online learning [21].

Successful participation and proper interaction during learning activities are essential for ensuring the efficiency and effectiveness of learning programs [22]. We further evaluated the perception of medical students regarding their participation in class activities during online learning. A noteworthy observation was a considerable number (~ 60%) of students exhibited the least engagement and interaction with both their classmates and teachers during their online sessions which might be because of no proper communication between the teachers and learners which was also reported by Manusov et al. [21]. In India, 80% of students could not participate actively in their online classes due to lack of facilities and communication gaps [23]. There are multiple factors including the availability and adequacy of technology resources impacting their ability to effectively engage with online educational content and platforms [24, 25]. Studies have highlighted additional difficulties faced by students that lead to the least interaction with colleagues and teachers, including the absence of on-campus interactions, challenges with collaborative group projects, and delays in professors’ response times in online learning [26]. In contrast to our results, a study has reported that proper usage of technology can lead to increased student engagement and interaction during online learning [27].

Moving further, students were asked to share their perceptions regarding the accessibility to learning resources and their institution’s preparedness regarding online teaching. Most of the participants were satisfied with the learning material provided to them but 65% were of the idea that online course was not well-structured as compared to traditional learning course used to be. Kheng et al. have reported the important parameters for the preparedness of institutions regarding online teaching which can be attributed to our institutes [28]. In our study, students reported poor development of their laboratory and clinical skills. Up to 80% of students were not satisfied with their learned skills during e-learning. In contrast, many studies have reported satisfaction of students regarding their skills development and institutional preparedness in e-learning [29–32]. A Libyan study has reported the successful development of Lab techniques in medical students during their first experience of online learning [33]. Furthermore, a meta-analysis reported improvement in skills learned by medical students through online learning [34].

In our study, we found 75% were satisfied with the structure of online exams which is in accordance with multiple studies. Previous studies have reported that students showed significant satisfaction and better performance with online examinations [35]. A study reported that students were satisfied with online examinations however their performance did not show any correlation to that [36]. A study by Milone et al. has also reported the satisfaction of students regarding online exams [37]. A UK-based study reported more satisfaction and less anxiety experienced in online exams as compared to traditional exam patterns [38]. A survey of dental students also found higher scores and satisfactory remarks with online exams [39]. In contrast, one of the studies has reported that students’ performance and scores were better with the traditional method as compared to online and they opted to appear in traditional method of exams in the future [40].

Our study has reported the least satisfaction of medical students regarding online learning. Eighty-one percent (81%) of our study participants were not satisfied with the online learning experience. A study from India reported more than 50% of students’ dissatisfaction with online learning which is in accordance with our results [41]. A study from Hamdard University India also reported an unsatisfactory survey report of medical students regarding online learning [42]. In contrast, multiple studies have reported a positive and satisfactory response from medical students regarding online learning. A study by Sujarwo et al. yielded a more positive response from medical students on online learning [43]. A study from Saudi Arabia reported a better outcome of e-learning and a satisfactory response from medical students regarding shifting learning towards the online COVID-19 pandemic [44]. A French study reported more than 60% of medical students satisfaction with online learning during COVID-19 and agreed to continue this after the pandemic era [45]. A large-scale survey of 30 medical schools in the UK revealed about 70% of students would choose to continue online learning in the future [46]. Multiple studies have shown a satisfactory response regarding online learning, most of those studies are however from developed countries where there is proper availability of resources, trained staff, and institutes are well prepared for online teaching. In LMICs such as Pakistan, where a significant majority of students face substantial barriers to internet access due to both technical limitations and financial constraints [47], the feasibility of achieving desired educational outcomes through online learning is greatly hindered.

## Conclusion

The survey’s outcomes reveal that students have shown their dissatisfaction towards e-learning which is the highest reported from any LMIC to date. Difficulty in connectivity, electricity issues, less interaction with colleagues and teachers, and issues with the structure of online courses were the most frequently reported problems by the students. These findings will serve as valuable insights for academic institutions striving to design more effective learning environments that enhance the overall educational experience for students.

## Data Availability

All data has been included in the study however it is available with the corresponding author and may be provided on request.
